# Annotations

**Published:** 1903-08-01

**Authors:** 


### Annotations.

### Dying Declarations.

One or two recent instances in the Courts of Law suggest that the rules under which dying declarations must be taken in order to be admissible as legal evidence are not generally appreciated. In one case, both a magistrate and a medical practitioner acted in express opposition to these rules, with the result that at the trial the judge ruled the declaration to be inadmissible. Such a conclusion may readily lead to a grave miscarriage of justice. It is necessary to remember that the Courts are very strict in reference to this class of evidence, and unless all the conditions have been fulfilled the declaration will not be received. The first of these conditions is that the person making the declaration undoubtedly believes himself to be dying, and has given up all hope of recovery. There must be "a hopeless expectation of impending death," and the declarant's conviction on this point must appear on the face of the declaration and must be expressed in terms which exclude all doubt, ambiguity, and qualification. Even such a phrase as "I think I shall not recover" has been held to be insufficient. The second condition is that the declaration be made voluntarily and not as the result of outside pressure or in response to questions. In the third place, the declaration must be phrased in the exact words of the dying man. If his statements on any point appear to be confused or obscure it is permissible to ask questions to try to make his meaning clear, but every such question, and the precise terms of each answer, must be placed, *verbatim et literatim*, on the record. The declaration when completed should be read over to the dying man, and, if possible, he should sign it, as should also any witnesses who may be present. The responsibility for all these details often falls on the medical attendant, and it is manifestly his duty to see that the declaration is not rendered useless by negligence or ignorance on his part. As legal evidence, dying declarations are limited to charges of murder or manslaughter; they cannot be put in evidence against any prisoner charged with a lesser offence, nor are they admissible in civil trials.

### The Causes of Cancer.

Mr. Jonathan Hutchinson, in a recent article, argues that local irritation, rather than parasitic influence, is the cause of the cancerous process. He submits in support of his thesis a series of facts which came under his observation during his recent tour in India. First, it is to be noted, that amongst the Mohammedans, all of whom are circumcised, cancer of the penis is almost unknown, whereas the Hindoos, who have long foreskins and are negligent of cleanliness, contribute frequent victims to this disease. Again, cancer of the lips is very rare, whilst the tongue, the inner surface of the cheeks, and the floor of the mouth are not uncommon sites for epitheliomatous growths. The suggested explanation is that while pipe smoking is very exceptional, the universal habit of chewing the betel leaf mixed with lime is a source of constant irritation to the whole of the inner surface of the mouth. Hence, whilst the lips escape, the buccal mucous membrane often suffers. In the third place, attention is directed to the frequency of epithelioma affecting the abdomen and inner surface of the thigh in the inhabitants of Kashmir. It appears that in the winter it is the almost universal habit for these people to carry under their clothes, for purposes of warmth, a portable earthen brazier known as a "kangri." This hangs against the lower part of the abdomen, and in the sitting posture is placed between the thighs. As a result of this practice burns are common, and it is believed that irritation so produced accounts for the development of epithelioma. Whatever may be the issue of research in the laboratory, such facts as the above certainly lend emphasis to the contention that, as the exciting cause of cancer, local irritation plays a leading role.

### Alcohol in Patent Medicines.

A contemporary advises American women to refuse to have their houses, barns, and fences disfigured with advertisements. Anyone who knows to what an extent the advertisement system prevails in America will agree that on grounds of æsthetics alone the plea is justifiable, but one of the arguments used has a far wider bearing. Many of the advertisements are those of patent medicines, and the writer says that some of these medicines contain a large proportion of alcohol, in ignorance of which women who are keen temperance workers permit them to be advertised on their premises. One woman who had refused to have an advertisement of beer painted on her husband's barn afterwards "gave her consent to the exploitation of a patent medicine which contained 38 per cent. more alcohol than did the beer." She did not know that the medicine was only another form of drink, but, as the writer asks, "how long are intelligent women going to allow themselves to be in ignorance of this curse of the patent medicine?"—"a curse," as he says, "which is far more deep-seated and hurtful than what is called the drink question." In England we are not often asked to give up our walls to the advertisement of medicines, but we certainly are asked to buy and use all manner of patent drugs with fancy names, warranted to cure every disease under the sun. How many of those who consume these concoctions know what is in them? They may be harmless—and perhaps useless—but it is equally possible that they contain alcohol, and that the feeling that they do one good, which those who swallow them are eager to claim, is no more than the exhilaration that comes from alcohol, while the notion that it is "medicine" they are taking and not "drink" is in itself an insidious temptation. If they knew its constituents they would not touch it, but women who are scrupulous to the last degree about every article of food that enters their household are reckless in buying any much advertised nostrum. Even if there be no alcohol in the medicine, it does not follow that it is harmless, the drug habit being as bad as the drink habit, whether the drinking be open or concealed. In short, medicine should not be taken except under the instructions of a doctor, who knows what he is prescribing.

